# The German System

**Published:** 1911-07-08

**Authors:** 


### THE GERMAN SYSTEM.

### III. THE PRESENT POSITION OF HOSPITALS IN GERMANY (*Continued*).

By Our Special Commissioner.

In the last article we dealt exhaustively with the conditions under which the German hospitals exist to-day, and we laid stress on the fact that " Private Hospitals " in the State statistical returns are not " Voluntary Hospitals " in our sense of the word, but are for the most part private nursing homes which are purely business undertakings to which belongs only so much philanthropic credit as attaches to similar institutions in this country. We would once more impress on our readers the fact that the voluntary hospitals in Germany are very few; the mass of semi-public institutions that still have a modicum of voluntaryism clinging to them are not analogous to our own public hospitals. It is true they have a democratic elected curatorium or board of managament in many cases, but every patient who enters a ward bed has to pay for his treatment or somebody has to pay for him. The institution, even when it is an endowed Stiftung, only helps the poorer middle class patient by charging him slightly less than does the State hospital; the really poor person is paid for by the municipality whether he enters a public or a semi- private hospital (in case there is no available bed in the municipal hospital), and this payment is according to a fixed scale, which has been drawn up by the local authorities, and under which the hospitals have certain privileges. The definition of a poor man, according to these regulations, is similar to that which obtains with us; the liability of the communal authority to defray his hospital expenses, conditioned by local by-laws in accordance with the Act, is clearly laid down by the *Reichsgesetz über den Unterstutzungswohnsitz vom* 6 *Juni* 1870, as modified by the Act of 1894. This Act prescribes that every North German is entitled to public assistance by the communal authorities in case he is himself unable to pay the costs of his treatment. Such assistance is given, under the Act, by district and provincial pauper boards.

### The Influence of Social Legislation on the Hospitals.

Amplifications of this Act were made in 1871 and 1891, and it is incontestable that this social legislation has had an enormous effect upon the development of the German hospital system. In fact, it is necessary to bear the *Unterstutzungswohnsitz* in mind when dealing with the influence of insurance legislation upon the growth of German hospitals. In a former article of this series we have tried to show what insurance legislation has done for the municipal hospitals of Germany, but it would be grossly unfair to ascribe all the development and progress that have taken place of recent years in the hospitals of the Empire solely to the influence of the Acts regulating invalidity, sickness and accident insurance. The social legislation has undoubtedly contributed a great deal to promote such expansion as has taken place, and we must never lose sight of the fact that purely medical and economic influences have reacted as powerfully upon the German hospitals as have the insurance and social legislative enactments.

### The Income of German Hospitals.

We lay stress on these points because there is an unfortunate tendency in some quarters to ascribe the progress that has been made in German hospitals solely to the influence of social and more particularly to insurance legislation. The Chancellor of the Exchequer's recent statement that the income of the German voluntary hospitals has largely increased, as the direct result of the introduction of State insurance is not borne out by an examination of such statistics as are available. That the average hospital income has increased since 1876 is undoubted, but such increase is due as much to the effect of the Act of 1870 as to the effect of the insurance laws themselves. We have already shown how far hospitals, both voluntary and municipal, in Germany have directly benefited through the payments made to them by insurance societies. The statistics for the " private hospitals " cannot be obtained, and there is no possibility, therefore, of showing in a tabulated statement what portion of their income has been derived from purely philanthropic sources and what from patients' payments. Mr. George's statement, it seems to us, is an arbitrary assertion, which is incapable of being substantiated or refuted by actual figure. When it is modified to the effect that the total income of all German hospitals has trebled during the four decades following the introduction of insurance legislation, no one will dispute it. The only point then is whether such increase is due to the insurance legislation primarily or whether (as is much more probably the case) it is the result of the beneficial legislation initiated by the *Unterstutzungswohnsitz* Act.

### Some Main Facts.

Let us once more repeat the main contentions which we have endeavoured to outline in these articles, and for the sake of clarity we may put them in the form of definite categorical statements, the truth of which will be admitted by anyone who has studied the literature and statistics.

1. " Private hospitals " in Germany are not voluntary hospitals but mainly nursing homes; other private hospitals are few in number, not more than a fourth of the total number of " private hospitals " registered, and are supported in the main by religious or semi-religious bodies.

2. Both private and public hospitals charge fees for the treatment of patients; in the case of poor patients these fees must be paid either by the insurance societies or clubs, or, if the patient is not insured, by the pauper boards.

3. Where a large voluntary hospital takes the place of a municipal or city hospital, whether it is a religious institution or not, the communal authorities very often make a definite grant to it.

4. Where an association, whether religious or not, desires to erect a private hospital, the municipality gives a grant towards the building of the institution, provided it is proposed to make it an "open" and not a closed (that is, private nursing home) institution.

5. A few strictly voluntary hospitals exist in the shape of richly endowed hospitals or as institutions owned by philanthropists, upon whom falls the burden of maintaining them. Hospital appeals, as we know them, are not the rule in Germany.

### Some Further Statistics of Revenue.

We have been asked to give a tabulation of the total income of such large private hospitals as may legitimately, by reason of the fact that they are open institutions, be considered to belong to the category of voluntary hospitals. This for the reason mentioned (that no statistics are available) it is impossible to do. In order to arrive at some definite conclusion regarding the effect of the insurance laws, for instance, upon the voluntary contributions to the German hospitals, it would be necessary to take the total income of all philanthropic bodies that support hospitals and to arrange those in tables showing the totals before and after the introduction of insurance legislation. These statistics are difficult, if not impossible, to obtain, and we have so far failed to secure any reliable evidence on which such a tabulation could be founded. The percentage increase in the voluntary contributions of such a society as the Frauenverien, which maintains several institutions under the Red Cross, does not show that there is any great difference between a decennium before and after the introduction of insurance legislation. The most that can be said is that such legislation does not appear to have had any appreciable influence upon the total yearly income of these societies.

In the useful handbook issued by Leineweber* an interesting tabulation of the income and expenditure of fifty-two of the largest German institutions is given, from which we take the following abbreviated table, giving the incomes of twenty-six hospitals.

Table showing total income of 26 large German Hospitals.

| | No. of Beds. | | Receipts from Endowments. | Direct State Grants. | Patients' Payments including Municipal Patien | Receipts from various sources, including Municipal Grants. | Total. |
|---|---|---|---|---|---|---|---|
| | Patients. | Staff. | Marks. | Marks. | Marks. | Marks. | Marks. |
| 1 | 563 | 202 | 1,880 | Nil | 377 426 | 26,708 | 406,014 |
| 2 | 578 | 112 | 10.891 | Nil | 254.861 | 6,884 | 272,637 |
| 3 | 402 | 78 | 2.866 | Nil | 133,782 | 16,035 | 252 649 |
| 4 | 1,594 | 680 | Nil | Nil | 647,129 | 25,852 | 672,981 |
| 5 | 732 | 224 | Nil | Nil | 397.516 | 11,973 | 409,490 |
| 6 | 763 | 230 | 1,163 | Nil | 275.506 | 111,565 | 389,585 |
| 7 | 220 | 48 | 225 | Nil | 29,839 | 1,167 | 31,232 |
| 8 | 662 | 333 | 26,603 | Nil | 475,513 | 3,285 | 505,302 |
| 9 | 854 | 160 | Nil | Nil | 499,826 | 24,607 | 525,433 |
| 10 | 1,200 | 414 | 6.175 | Nil | 765,657 | 7,000 | 778.557 |
| 11 | 520 | 159 | 59,490 | Nil | 905.361 | 6,[illegible]00 | 270.870 |
| 12 | 500 | 86 | 7,055 | Nil | 260.026 | 34,284 | 301,646 |
| 13 | 586 | 104 | 8,181 | Nil | 331.996 | 8,264 | 351,782 |
| 14 | 1.258 | 216 | 5,161 | Nil | 722,074 | 13,596 | 748,085 |
| 15 | 912 | 315 | Nil | Nil | 651.513 | 145.000 | 796.736 |
| 16 | 520 | 120 | 3,765 | Nil | 321,532 | 8.205 | 333,908 |
| 17 | 1,125 | 543 | Nil | Nil | 906,132 | 104,540 | 1,012,436 |
| 18 | 1,521 | 476 | Nil | Nil | 1,049,740 | 28.923 | 1,069,799 |
| 19 | 1.500 | 350 | 19.563 | 50,000 | 938,773 | 149.374 | 1,157,699 |
| 20 | 1,320 | 200 | 142.299 | 6.301 | 1,187,598 | 24,113 | 1,361,329 |
| 21 | 660 | 120 | 5.320 | 6,857 | 287.059 | 51,816 | 348.762 |
| 22 | 544 | 90 | Nil | 15.239 | 284.778 | 5,799 | 442,969 |
| 23 | 662 | 135 | 6,623 | 15,000 | 519,352 | 779,843 | 1,319,915 |
| 24 | 254 | 99 | 46.689 | 30,000 | 86,379 | 2,484 | 165,587 |
| 25 | 225 | 71 | 23,781 | 7,000 | 23,162 | 34,723 | 158,666 |
| 26 | 550 | 122 | 2,407 | 95.738 | 319,180 | 73,391 | 490,771 |

We have compiled a list of the voluntary German hospitals from Leineweber's *Adressbuch*, which contains much useful information relative to the condition, number of beds, boards of management, etc., of these institutions, but this is too long to be included in these articles, and we must refer readers to the volume quoted.

### Penalising the Non-Paying Patient.

It is further interesting to note that under section 3 of the Franchise Act of May 1859, persons who have availed themselves of pauper hospital treatment are *ipso facto* deprived of their voting rights. The *Deutsche Verein für Armenpflege und Wolhthätigkeit* has laboured in a praiseworthy fashion to get this clause amended, but so far without success. The short-sighted policy of the State in enforcing such a handicap has often been pointed out, and hospital workers in the Empire have discussed ways and means of altering it. It has been suggested that the poor scale in the communal hospitals (wards of the third class) should be raised, just as it has been proposed that the scale for insured patients should be grounded, not on an arbitrary average of the cost per bed in all hospitals, but on the "interest figure" of each bed—in other words, that beds should be reserved for insurance patients at a rate equal to the annual interest of the capital necessary to endow a bed in perpetuity—a figure which will of course vary with the size and equipment of the institution. The point is a very interesting one for us at the present moment. If we are going to charge our insured patients we must have some basis on which to found an adequate charge, and the suggestion that this should be the capital expenditure on a bed for perpetual endowment (not the arbitrary £1,000, which it is now generally admitted is quite insufficient, but a fair average, which would not entail any loss, is well worth consideration.

### The Voluntary and Semi-Voluntary Hospitals.

We may now devote some attention to the larger private institutions, which are closest to our own voluntary hospitals. These belong for the most part to religious denominations, to sisterhoods or brotherhoods, to philanthropic societies, and to the Red Cross Association. Others, belonging to private philanthropists, wholly endowed or supported by them, to firms, to consumption sanatorium associations, and to insurance societies, hardly fall under this category and may be omitted from this article.

*Hospitals belonging to Religious Denominations.*—These are the Catholic and Evangelical hospitals, some of them with as many as 600 beds, and others, again, mere cottage hospitals with a few beds only. They are managed by elective boards, at the head of which is usually the local pastor or priest, and are supported out of the church funds, each parish maintaining its own hospital. Patients' payments are a lucrative source of income, as the establishments are most economically managed. The staff is paid by the church, but the nurses, who mostly belong to Church sisterhoods, and a few honorary surgeons and physicians of repute give their services gratuitously. Poor patients are paid for by the municipality, which usually also gives a yearly contribution towards the upkeep of the hospital. Voluntary contributions are collected locally and elsewhere, but are small in amount.

*Hospitals belonging to Sisterhoods and Brotherhoods.*—There are many of these, both Catholic and Evangelical. The following are the chief Catholic sisterhoods and brotherhoods which possess their own institutions: Brothers of St. Alexius (12 hospitals); Brothers of Charity

* *Taschenbuch für den wirtschaftlichen und verwaltungstechnischen Krankenanstalts-Betrieb.* Zweiter Jahrgang, 1911. Herausgegeben von B. Zeidler.

of St. John, founded in the sixteenth century in Spain (22 hospitals); Brothers of Charity of Montabaur, founded in 1852 (18 centres); Brothers of Charity of St. John of Trier (25 centres); Brothers of St. Francis (10 centres); Sisters of Charity of St. Augustine, with more than 50 centres; Sisters of Charity of St. Borromaeus, founded in 1652, possessing nearly 200 centres and a large number of hospitals, chiefly maternity institutions; Sisters of the Holy Heart, founded in 1866 (8 centres); Poor Sisters of Jesus, founded in 1852 (more than 100 centres; a few hospitals only); Dominican Sisters of St. Catherine, who are Sisters of the Third Dominican Order (34 centres); Sisters of Elizabeth under the rule of St. Francis, founded in 1395, possessing about 20 institutions; Sisters of St. Francis of the Holy Family, founded in 1875 (2 hospitals); Poor Sisters of St. Francis (1 maternity hospital); Sisters of St. Francis of Waldbreitbach (general nursing; no special hospital as yet); Sisters of St. Francis, of the Third Order (7 maternity hospitals); Sisters of the Atonement, founded in 1835 (statistics not available); Grey Sisters of St. Elizabeth (several hospitals); Sisters of St. Joseph, founded 1845 (one hospital); Sisters of St. Catherine (6 centres); Sisters of the Holy Child, founded in 1807 (7 centres); Sisters of Charity of St. Clement (84 centres, with several hospitals); Sisters of the Holy Cross (nursing sisterhood, with no hospitals of their own); Sisters of St. Mary (1 hospital); Sisters of the Immaculate Conception (1 hospital); Sisters of St. Mary Magdalene (1 hospital); Sisters of St. Vincent, founded in 1633 (12 hospitals).

The following Evangelical sisterhoods possess their own hospitals; they are all grouped together under the general term Diakonissen Krankenhäuser: Kaiserwerth; Berlin; Strassburg; Dresden; Bethanien (Berlin); Breslau; Königsberg; Ludwigslust; Karlsruhe; Neuendettelsau; Stuttgart; Augsburg; Halle; Darmstadt; Speyer; German Samaritans at Kraschnitz; Henrietta Order in Hanover; Rotenburg; Danzig; Kassel; Mitau; Lazarushaus (Berlin); Posen; Frankenstein; Riga; Reval; Helsingfors; Altona; Bremen; Salem-Stift at Stettin; Sarepta at Bielefeld; Bethanien at Stettin; Braunschweigh; Frankfort; Flensburg; Potsdam; Paul Gerhard Stift (Berlin); Ingweiler; Niesky; Mannheim; Württemberg; Elizabeth Children's Hospital (Berlin); Kreuzberg; Kreuznach; Oldenburg; Witten am Rhein; Lindenau; Eisenach; Frankfurt am Oder; Wiesbaden; Detmold; Grunberg; Magdeburg; and Leipsig.

The hospitals belonging to these institutions are kept up by contributions obtained not only from Germany, but from all parts of the world, and definite grants are made by the congregations in support. Fees are charged in most cases. The Catholic orders as well as the Evangelical occupy themselves very largely with nursing, maternity (district) work, and with education, so that it is impossible to state definitely what part of the contributions are devoted to the upkeep of hospitals.

The Red Cross Association for Germany has its centre in Berlin, and has an unusually large number of voluntary aid associations affiliated to it. It possesses a number of fine and modern hospitals, which are mainly supported out of the general fund of the Association. The Women's Association of Baden, an affiliated society, owns and controls a large hospital with 125 beds at Karlsruhe. The Association possesses some 50 filials with their own hospitals.

The two great nursing and semi-religious orders in Germany are the Brandenburg Johanniter Orden, founded in 1852 by Frederick William IV., an Evangelical order which possesses more than 50 hospitals, and the Malteser Orden, founded in 1867, a Catholic organisation with 10 hospitals Three good hospitals in Bavaria are owned by the Bavarian Hausritter Orden of St. George.

With the exception of these institutions, the private hospitals in Germany have no claim to be considered as voluntary hospitals dependent for their maintenance on voluntary charity. They are purely business undertakings, and although they are to a certain extent under the control, so far as the concession and sanitary inspection is concerned, of the municipal and provincial authorities, they are quite independent and not in any sense of the word public or semi-public institutions. That they have increased so hugely during the last twenty years is not primarily and entirely the result of the Insurance Acts, but purely due to the fact that with the rapid development of medicine and surgery in Germany it is becoming increasingly recognised that patients who are suffering from certain conditions are best treated in an institution and not at home. The fact that there is Government inspection, registration, and supervision has inspired the public with a certain amount of confidence in these private nursing homes, which are indeed for the most part admirably managed, equipped, and arranged. The shortcomings of our own system of private nursing homes have too often been dealt with in the columns of The Hospital to need reiteration here, but it is worth bearing in mind that it is highly probable so much popularity would not have accrued to the German private clinics had they not been placed under public inspection as ours ought to be.

[We have been asked to include in these articles particulars regarding the payments made to the staff of hospitals which receive State or municipal aid. While it seems more convenient to deal with this subject in a special chapter, it may be mentioned here that where State or municipal aid is given, payment of the medical staff is equally the rule. As a general rule the salaries of the staff are calculated on a fixed scale, and the number of beds, number of voluntary assistants, and general income of the institution are taken into account. It must be remembered that in many cases the principal members of the staff are professors or consultants of wide reputation in their specialty. We hope to devote a special article to the consideration of the whole question.—Ed., The Hospital.]

